# CCR5 Controls Immune and Metabolic Functions during *Toxoplasma gondii* Infection

**DOI:** 10.1371/journal.pone.0104736

**Published:** 2014-08-13

**Authors:** Giuliano Bonfá, Luciana Benevides, Maria do Carmo Souza, Denise Morais Fonseca, Tiago Wilson Patriarca Mineo, Marcos Antônio Rossi, Neide Maria Silva, João Santana Silva, Cristina Ribeiro de Barros Cardoso

**Affiliations:** 1 Departamento de Bioquímica e Imunologia, Faculdade de Medicina de Ribeirão Preto, Universidade de São Paulo, Ribeirão Preto, São Paulo, Brazil; 2 Departamento de Patologia, Faculdade de Medicina de Ribeirão Preto, Universidade de São Paulo, Ribeirão Preto, São Paulo, Brazil; 3 Departamento de Análises Clínicas, Toxicológicas e Bromatológicas, Faculdade de Ciências Farmacêuticas de Ribeirão Preto, Universidade de São Paulo, Ribeirão Preto, São Paulo, Brazil; 4 Instituto de Ciências Biomédicas, Universidade Federal de Uberlândia, Uberlândia, Minas Gerais, Brazil; Charité, Campus Benjamin Franklin, Germany

## Abstract

CCR5, an important receptor related to cell recruitment and inflammation, is expressed during experimental *Toxoplasma gondii* infection. However, its role in the immunopathology of toxoplasmosis is not clearly defined yet. Thus, we inoculated WT and CCR5^-/-^ mice with a sub lethal dose of the parasite by oral route. CCR5^-/-^ mice were extremely susceptible to infection, presenting higher parasite load and lower tissue expression of IL-12p40, IFN-γ, TNF, IL-6, iNOS, Foxp3, T-bet, GATA-3 and PPARα. Although both groups presented inflammation in the liver with prominent neutrophil infiltration, CCR5^-/-^ mice had extensive tissue damage with hepatocyte vacuolization, steatosis, elevated serum triglycerides and transaminases. PPARα agonist Gemfibrozil improved the vacuolization but did not rescue CCR5^-/-^ infected mice from high serum triglycerides levels and enhanced mortality. We also found intense inflammation in the ileum of CCR5^-/-^ infected mice, with epithelial ulceration, augmented CD4 and decreased frequency of NK cells in the gut lamina propria. Most interestingly, these findings were accompanied by an outstanding accumulation of neutrophils in the ileum, which seemed to be involved in the gut immunopathology, once the depletion of these cells was accompanied by reduced local damage. Altogether, these data demonstrated that CCR5 is essential to the control of *T. gondii* infection and to maintain the metabolic, hepatic and intestinal integrity. These findings add novel information on the disease pathogenesis and may be relevant for directing future approaches to the treatment of multi-deregulated diseases.

## Introduction

CCR5 is a CC chemokine receptor expressed in several cell types including macrophages, dendritic cells, NK, NKT and T cells [1]–[4]. The main chemokine ligands of CCR5 are CCL3 (MIP-1α), CCL4 (MIP-1β) and CCL5 (RANTES), which play an important role in cell recruitment to the local of inflammation [3], [5]. These CCR5 ligands are extensively expressed during several inflammatory processes [6]–[9], including parasite infections [10], [11], such as in toxoplasmosis [12], [13].

Toxoplasmosis, a widespread infection throughout world, is caused by the intracellular parasite *Toxoplasma gondii*. Its incidence in humans varies between 9% in the USA and can reach 72% in some regions of Latin America countries, like Brazil [14]–[16]. The intense intestinal inflammation observed in C57BL/6 mice after oral experimental *T. gondii* infection share morphological and histological characteristics with human inflammatory bowel disease (IBD), such as massive necrosis, shortening of the villi and concentrated influx of leukocytes into the lamina propria (LP), resulting in overall loss of intestinal epithelial architecture [17]–[19]. This pathology is mediated by a robust Th1 cell response, mainly characterized by an influx of CD4^+^ T cells and exacerbated production of IFNγ, TNF and nitric oxide [20], [21]. If Th1 immune response is not controlled, oral infection of C57BL/6 mice culminate in a lethal ileitis a few days post-infection [18], [21], [22]. However, if Th1 immune response is controlled, there is survival of the host and the infection becomes chronic, as observed in BALB/c mice [20] or in C57BL/6 mice infected with a relatively low dose of *T. gondii* [23]. Indeed, parasite strain, inoculation route, dose of inoculum, host background and immunosuppression will dictate whether gut damage or protection occurs [24].

During *T. gondii* infection, toll like receptors (TLRs) and chemokine receptors may function together in APC activation and IL-12 production [25]–[27], thus determining the infection outcome. Partial IL-12 production comes from the interaction between chemokines such as CCL3, CCL4 and CCL5, produced by immune and epithelial intestinal cells after *T. gondii* infection, with CCR5 [4], [22]. Cyclophilin 18 (C-18), a protein produced by the parasite and found in the soluble antigen fraction of *T. gondii* (STAg), can also be recognized by CCR5 and induce APC activation with consequent IL-12 production [25]. These chemokines and chemokine-like molecules can induce the migration of CD8^+^ lymphocytes to intraepithelial region of small intestine by interaction with CCR5 and this signaling may be essential to the control of the exacerbated inflammatory response induced by the parasite through TGF-β production [12], [28]. Moreover, in other disease models or infection CCR5 also modulate the migration and suppressor function of regulatory T cells and may interfere in pathogen control or immune response regulation [6], [10], [29]. Therefore, the expression of CCR5 in T cells is related to both pro- and anti-inflammatory functions and, although it is known that this receptor is expressed during infection by *T. gondii*, its role in the immunopathology on specific sites of infection is not clearly defined yet. Accordingly, to better understand the immune and metabolic roles of CCR5 during acute infection with *T. gondii*, we used CCR5 genetically deficient (CCR5^-/-^) mice orally infected with sub lethal parasite inoculum. We confirmed that in the absence of CCR5, mice were extremely susceptible to infection. This augmented susceptibility to infection was due to higher parasite load, associated with increased intestinal and hepatic tissue damage, along with an outstanding accumulation of neutrophils in the ileum. Finally, this study pointed to CCR5 as an essential molecule involved in the maintenance of host homeostasis during *T. gondii* infection.

## Materials and Methods

### Animals

Female C57BL/6 wild type (WT) and CCR5-deficient (CCR5^-/-^) mice, with 8–10 weeks of age were used. CCR5^-/-^ mice were purchased from The Jackson Laboratory (Bar Harbor, ME) All mice were bred and maintained in small groups inside isolator cages with light/dark cycle of 12 hours, besides food and water ad libitum, in the animal housing facility of School of Medicine of Ribeirão Preto, University of São Paulo. All the experiments were developed in accordance to ethical principles in animal research adopted by Brazilian Society for Laboratory Animal Science and approved by the Ethics Committee on Animal Experiments, School of Medicine of Ribeirão Preto (Permit Number: 200/2009).

### Parasites, experimental infection and PPARα agonist treatment

The experimental infection was performed with *T. gondii* tissue cysts of the ME-49 stain, maintained by serial passages in C57BL/6 mice [23]. Briefly, thirty to forty days after infection, tissue cysts were harvested from the brain, which were homogenized in 2 ml of PBS (pH 7.2), counted and diluted. CCR5^-/-^ and WT mice were orally infected with 5 cysts of the parasite in 0.2 ml of PBS, a condition that leads to 75–100% survival in C57BL/6 mice. Mortality (7–10 animals by group), immunological and biochemical parameters (3–4 animals/group) were evaluated.

For administration of Gemfibrozil (GEM) (Sigma-Aldrich, St. Louis, MO), a stock solution of 100 mg/ml was prepared by dissolving the drug in ethanol 100%. The stock solution was diluted in carboxymethylcellulose (CMC) to 0.75% and the infected mice were treated orally for 7 days, beginning in the first day of infection, with 100 mg/kg/day of the drug in a total volume of 0.2 ml. Infected control animals were treated with an equivalent amount of ethanol and CMC vehicle. For histological and biochemical evaluation, liver and blood of animals were collected and stored appropriately according to each experimental protocol.

The survival curve, which was performed with the minimal number of mice as possible, was necessary to define the correct time point for sample collection. For that, humane endpoints (such as daily clinical evaluation) were considered to define the best moment for euthanasia, which was later performed at day 8 post-infection using CO_2_ chamber. Furthermore, the infection was performed with a sub lethal dose of a non- or low-virulent parasite strain (ME-49). All these efforts together were made to minimize mice suffering, along with the use of the lower number of animals as possible in each subsequent experiment.

### DNA extraction and quantification of tissue parasite burden

Fragments of small intestine and liver were collected at day 8 post-infection and stored in a dry tube at -70°C. Genomic DNA was extracted from approximately 30 mg of tissue or from 10^7^ *T. gondii* tachyzoites (RH strain) using the Kit illustra Tissue & Cells GenomicPrep (GE Healthcare, Freiburg, Germany). DNA was quantified in a spectrophotometer (Thermo Scientific NanoDrop 1000, Wilmington, DE) and parasite burden was assessed by quantitative PCR (qPCR) using SYBR Green (Life Technologies, Carlsbad, CA, USA), from 0.1 µg of each sample. The reaction was performed in a Sequence Detection System ABI 7000 (Life Technologies) with the following parameters: 2 min. at 50°C, 2 min. at 95°C, forty cycles of 15 sec. at 95°C, 30 sec. at 58°C and 30 sec. at 72°C, beyond a dissociation step, with temperature ranging from 60 to 95°C. Amplification of parasite DNA was performed using primer pairs specific for the conserved *T. gondii* B1 gene (sense: 5′-TTC AAG CAG CGT ATT GTC GA-3′ and antisense: 5′-CAT GAA CGG ATG CAG TTC CT-3′ - MWG Oligo Synthesis Report, Miami, FL), that is found in all known parasite strain. The results were obtained based on standard curve made with graded concentrations of parasite DNA.

### RNA extraction and real time quantitative PCR (qPCR)

RNA was extracted from ileum and liver of WT and CCR5^-/-^ mice harvested by a method that combine TRIzol reagents (Life Technologies) and a partial utilization of extraction Kit illustra RNA spin Mini (GE Healthcare) following manufacturer's instructions. After extraction, RNA quantity, purity and quality were determined by a spectrophotometer (Thermo Scientific NanoDrop 1000). Complementary DNA (cDNA) was synthesized using 2 µg of RNA and reverse transcriptase SuperScript III (Life Technologies) on termocycler PTC-100 (MJ Research, Watertown, NY). The cDNA was amplified in a qPCR using SYBR Green (Life Technologies) and gene-specific primers in a real-time PCR thermocycler. The primers sequences used for qPCR are presented in the Table 1. The reaction followed the parameters: 2 min. at 50°C, 2 min. at 95°C, forty cycles of 15 sec. at 95°C, 30 sec. at 58°C and 30 sec. at 72°C, beyond a dissociation step, with temperature ranging from 60 to 95°C. Ct data (cycle threshold) were normalized to the expression of reference gene (GAPDH) and analyzed using 2^-ΔΔCt^ method where ΔΔCt  =  ΔCt sample – ΔCt control sample, in which ΔCt  =  Ct (studied gene) – Ct (reference gene).

**Table 1:** Primer sequences used in qPCR.

Primers	Sequences
GAPDH	sense: 5′-TTCAAGCAGCGTATTGTCGA-3′
	antisense: 5′-CATGAACGGATGCAGTTCCT-3′
IL-12p40	sense: 5′-AGCACCAGCTTCTTCATCAGG-3′
	antisense: 5′-GCGCTGGATTCGAACAAAG-3′
IFN-γ	sense: 5′-TTTAACTCAAGTGGCATAGATGTGG-3′
	antisense: 5′-TGCAGGATTTTCATGTCACCAT-3′
TNF	sense: 5′-AGGGATGAGAAGTTCCCAAATG-3′
	antisense: 5′-GGCTTGTCACTCGAATTTTGAGA-3′
IL-1β	sense: 5′-TGACAGTGATGAGAATGACCTGTTC-3′
	antisense: 5′-TTGGAAGCAGCCCTTCATCT-3′
IL-17	sense: 5′-TGCCCTCCACAATGAAAAGA-3′
	antisense: 5′-AACACGAAGCAGTTTGGGAC-3′
IL-6	sense: 5′-TTCCTACCCCAATTTCCAAT-3′
	antisense: 5′-CCTTCTGTGACTCCAGCTTATC-3′
IL-4	sense: 5′- AAGAGCATCATGCAAATGGA-3′
	antisense: 5′- TTAAAGCATGGTGGCTCAGTAC-3′
CCL3	sense: 5′- TTCTGCTGACAAGCTCACCCT-3′
	antisense: 5′ATGGCGCTGAGAAGACTTGGT-3′
CCL4	sense: 5′-CCTGACCAAAAGAGGCAGACA-3′
	antisense: 5′-AGCAAGGACGCTTCTCAGTGA-3′
CCL5	sense: 5′-TTCCCTGTCATCGCTTGCTCT-3′
	antisense: 5′-CGGATGGAGATGCCGATTTT-3′
CCR1	sense: 5′-GTGGGCAATGTCCTAGTGATT-3′
	antisense: 5′-GGTAGATGCTGGTCATGCTTT-3′
CCR2	sense: 5′-TTGATCTTTCCTTCTGCTTT-3′
	antisense: 5′-TCACTACTTTTAGCCTTCTGCT-3′
CCR3	sense: 5′-GCTCTCTGGATTGAAGTGTGCA-3′
	antisense: 5′-AAGTATCACGTCCACCACCTGG-3′
CCR4	sense: 5′-CGATTCCAAAGATGAATGCCA-3′
	antisense: 5′-TCCCCAAATGCCTTGATACC-3′
CCR5	sense: 5′-TGCACAAAGAGACTTGAGGCA-3′
	antisense: 5′-AGTGGTTCTTCCCTGTTGGCA-3′
CXCR3	sense: 5′-AACGTCAAGTGCTAGATGCCT-3′
	antisense: 5′-TCTCGTTTTCCCCATAATCG-3′
iNOS	sense: 5′-CGAAACGCTTCACTTCCAA-3′
	antisense: 5′-TGAGCCTATATTGCTGTGGCT-3′
Foxp3	sense: 5′-ACAACCTGAGCCTGCCACAGT-3′
	antisense: 5′-GCCCACCTTTTCTTGGTTTTG-3′
GATA-3	sense: 5′-AGGAGTCTCCAAGTGTGCGAA-3′
	antisense: 5′-ATGGAATGCAGACACCACCT-3′
PPARα	sense: 5′-TCAATGCCTTAGAACTGGATGA-3′
	antisense: 5′-CCGATCTCCACAGCAAATTATA-3′
PPARγ	sense: 5′ TGAGATCATCTACACGATGCTG-3′
	antisense: 5′-AGGAACTCCCTGGTCATGAA-3′
ROR-γt	sense: 5′-TGGAAGATGTGGACTTCGTTT-3′
	antisense: 5′-TGGTTCCCCAAGTTCAGGAT-3′
T-bet	sense: 5′-CCCCTGTCCAGTCAGTAACTT-3′
	antisense: 5′-CTTCTCTGTTTGGCTGGCT-3′

### Treatment of mice with anti-Gr-1 antibody

For depletion of neutrophils *in vivo*, mice were treated intraperitoneally with ammonium sulfate-precipitated RB6 (clone 8C5) culture supernatant at 500 µg/dose, one day prior to infection and then one and three days after *T. gondii* infection. Protein concentration was determined by Bradford protein assay (Thermo Scientific, Rockford, USA). Control animals received a similar dose of ammonium sulfate-precipitated whole rat IgG.

### Histological analysis

Fragments of small intestine and liver of WT and CCR5^-/-^ mice were harvested on day 8 post-infection, fixed in 10% buffered formalin and paraffin processed. Tissue sections of 5 µm thickness were mounted on slides and stained with hematoxylin and eosin. The images were acquired with a digital camera (Leica DC300F, Leica Microsystems AG, Heerbrugg, Switzerland) coupled to a Leica DMR microscope (Leica Microsystems) and a computer, in a magnification of 200 and 400X, for histological analysis. The histological score was analyzed as previously described [30] with some adaptations. Briefly, the parameters: intensity of lamina propria (LP) inflammatory infiltration, thickening of LP, destruction of the villi and necrosis were evaluated by intensity, represented as arbitrary units ranging of 0 (less intense or absent) to 5 (highly intense) for each parameter. The histological final score is a sum of each parameter for each mouse.

To identify steatosis, the livers of WT and CCR5^-/-^ mice were collected from naïve and infected mice and frozen in OCT medium (Sakura Finetek, Inc., Torrance, CA). Five µm thick tissue sections were obtained with the aid of a cryostat. The slides were fixed in 10% buffered formalin for 10 minutes, washed immediately in 3 changes of distilled water, air dried for 3 minutes, placed in 100% propylene glycol for 5 minutes and stained in pre-warmed Oil Red O solution for 8–10 minutes in 60°C oven. The sections were differentiated in 85% propylene glycol solution for 5 minutes, washed in 2 changes of distilled water and stained with hematoxilin. The slides were mounted with coverslips in aqueous medium containing glycerin and gelatin. The amount of lipids was quantified using dedicated software (Qwin Software, Leica) in conjunction with a microscope, video camera and an online computer in which lipid images were visualized as red-orange staining.

### Biochemical quantification of triglycerides and transaminases

To assess the magnitude of the injury in the liver of animals infected with *T. gondii*, the sera of WT and CCR5^-/-^ mice with 8 days of infection were submitted to determination of triglycerides and the activity of alanine aminotransferase (ALT) and aspartate aminotransferase (AST). Serum triglycerides were determined by colorimetric Enzymatic Trinder Method [31], following the manufacturer's standards (Labtest, Minas Gerais, Brazil). The concentration of ALT and AST in serum were determined by the colorimetric reaction of Reitman Frankel [32], following the manufacturer's standards (Labtest).

### Isolation of leukocytes and flow cytometry analysis

Lamina propria (LP) leukocytes were isolated on day 8 post-infection and identified according to the expression of the following markers: CD3, CD4, CD8 and NK1.1, detected by antibodies conjugated to PE, FITC or PE-Cy7 (BD Biosciences, San Diego, USA). The leukocytes from the small intestine were isolated using the method described by GUY-GRAND (1978) with some modifications [33]. Briefly, fragments of the small intestine were cut into small pieces in PBS containing 3 mM EDTA, stirred for 20 minutes, the cells were centrifuged, the supernatant was discarded and RPMI-1640 with 1% FBS, 1 mM EGTA and 1.5 mM MgCl_2_ was added to the sediment. After stirring, gut fragments were digested in 50 µg/ml of liberase TL (Roche, Mannheim, Germany) in incomplete RPMI medium. The resulting cells were filtered on nylon wool and centrifuged in Ficoll gradient. Viability was accessed by trypan blue exclusion and cells counted in a Neubauer chamber. The cells were washed and resuspended in PBS with the addition of BSA (1%), followed by Fc blockage and incubation with specific antibodies. After washing, all cells were fixed in PBS with 1% of formaldehyde, acquired on flow cytometer (FACSCantoII - BD Biosciences) and analyzed by FlowJo software (Tree Star, Inc., Ashland, OR) according to forward and side scatter dot plots.

### Immunohistochemical analysis for neutrophil detection

To identify the presence of neutrophils in gut and liver inflammation, immunohistochemistry was performed using a rat monoclonal antibody against Ly6B (mAb 7/4; Abcam, Cambridge, MA). Sections of deparafinized ileum and liver samples were subjected to antigenic retrieval in microwave oven and then incubated for 30 minutes at 37°C in PBS/1% bovine serum albumin (BSA; Sigma-Aldrich), followed by overnight incubation at 4°C with the primary antibody diluted 1∶50 in PBS/1% BSA. Secondary biotinylated antibody and streptavidin conjugated to peroxidase was used to improve the sensitivity of the assay (LSAB, Dako Cytomation, Carpinteria, CA). The reaction was visualized by incubating the sections with 3,3-diaminobenzidine tetrahydrochloride (DAB, Zymed Laboratories, Inc., San Francisco, CA). Control slides were incubated with non-immune rat serum. Counterstaining with hematoxylin was made to evidence the nuclei and the images were acquired as in histological analysis procedures.

### Statistical analysis

The Kaplan-Meier method was used to compare survival of animals and the mortality data of the experimental groups. To analyze data with parametric distribution we performed Student's t test or ANOVA with Tukey post-test. Data that did not assumed a normal distribution were analyzed with Mann Whitney or Kruskal Wallis Test with Dunn's Multiple comparison post test. Statistical analysis and graphics were performed using GraphPad Prism version 5.0 (GraphPad Software, San Diego, CA). All values were considered significant when P<0.05.

## Results

### CCR5^-/-^ mice are highly susceptible to *T. gondii* infection

**Figure 1:**
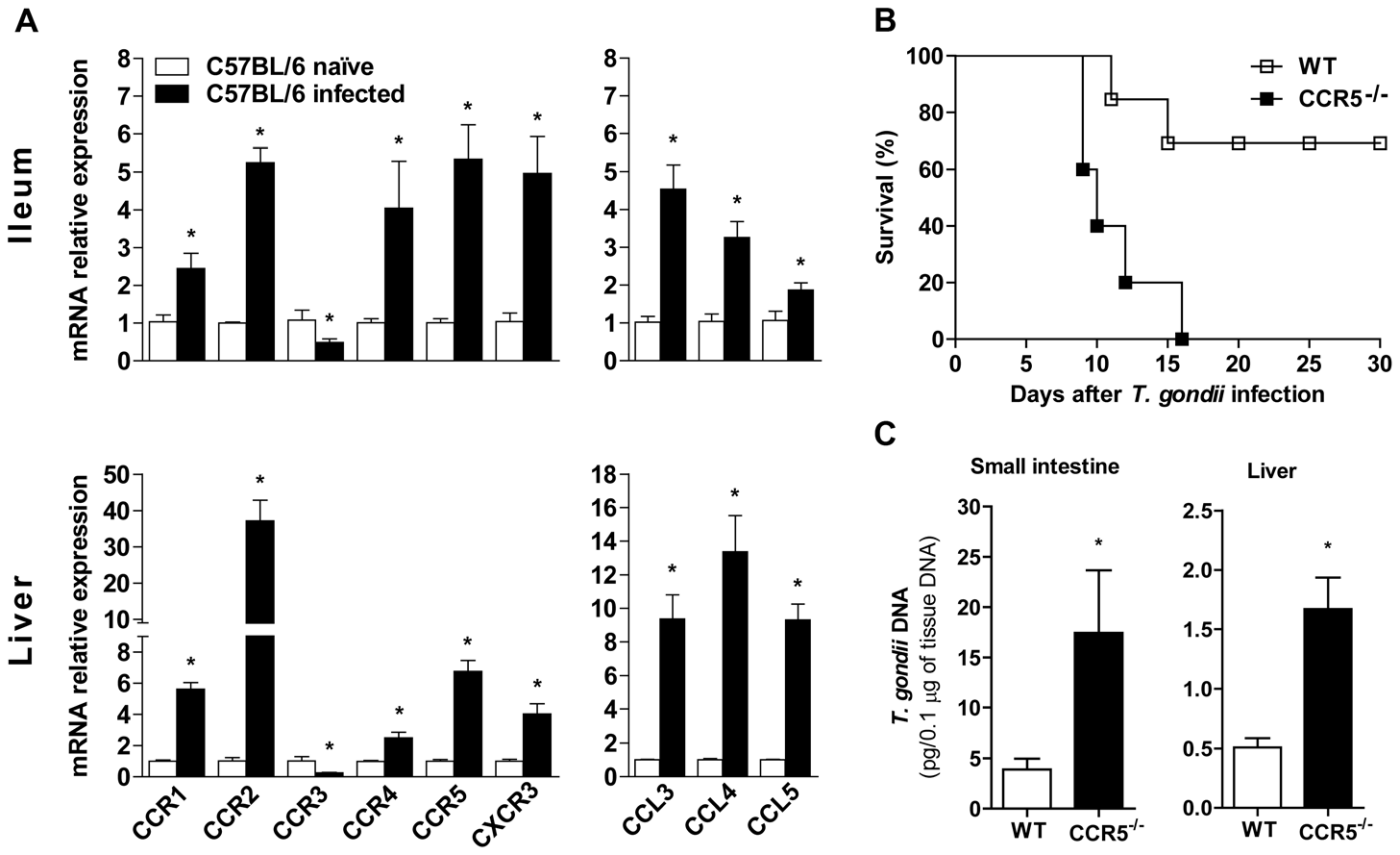
Chemokine and chemokine receptors expression after infection and CCR5^-/-^ mice susceptibility to *Toxoplasma gondii*. C57BL/6 (WT) mice were infected with 5 cysts of ME-49 strain of *T. gondii*. At day 8 pi, ileum and liver RNA were extracted and levels of CCR1, CCR2, CCR3, CCR4, CCR5, CXCR3, CCL3, CCL4 and CCL5 mRNA (A) were determined by real-time quantitative PCR (qPCR). WT and CCR5^-/-^ mice were infected and evaluated for survival until day 30 pi (B). At day 8 pi, small intestine and liver (C) were harvested, total DNA extracted and the tissue parasitism determined by qPCR based on curve with *T. gondii* DNA. * p<0.05 compared to WT naïve mice. Data are presented as mean ± SEM of three to seven mice per group and are a representative of at least two independent experiments.

As migration of leukocytes through chemokine-receptor interaction is essential to the early host defense mechanism in the pathogen control, we first determined the chemokine profile and receptors expressed in C57BL/6 mice on day 8 post-infection with *T. gondii*. We observed an increase in the expression of CCR1, CCR2, CCR4 and CXCR3 in the ileum and liver of infected mice (Figure 1A), as reported previously by our research group and others [12], [22], [23]. In addition, a concomitant down regulation of the Th2-related receptor CCR3 was observed in *T. gondii* infected mice, in accordance to the fact that eosinophils, mast cells or basophils are not the main leukocytes required for the control of this intracellular parasite. In contrast, there was a striking expression of the Th1-related chemokine receptor CCR5 in the ileum and liver of infected mice, as well as the chemokine ligands for this receptor CCL3 (MIP-1α), CCL4 (MIP-1β) and CCL5 (RANTES) at 8 days post infection (Figure 1A). Therefore, since there was a remarkable expression of CCR5 and its ligands in the *T. gondii* target tissues, we next assessed the relevance of this receptor to parasite control by infecting CCR5 deficient (CCR5^-/-^) mice with a sub lethal dose of this protozoan. The data reveal that CCR5^-/-^ mice showed accentuated susceptibility to infection, presenting 100% mortality until 16 days after *T. gondii* inoculation (Figure 1B), whereas 70% of WT animals survived until day 30 post infection. In addition, the small intestine and liver of CCR5^-/-^ mice showed higher parasite DNA in comparison to WT animals (Figure 1C). These results indicated that CCR5^-/-^ mice are highly susceptible to oral infection with *T. gondii* with an elevated parasite load in affected organs.

### Imbalance in the immune response regulation in *T. gondii*-infected CCR5^-/-^ mice

**Figure 2:**
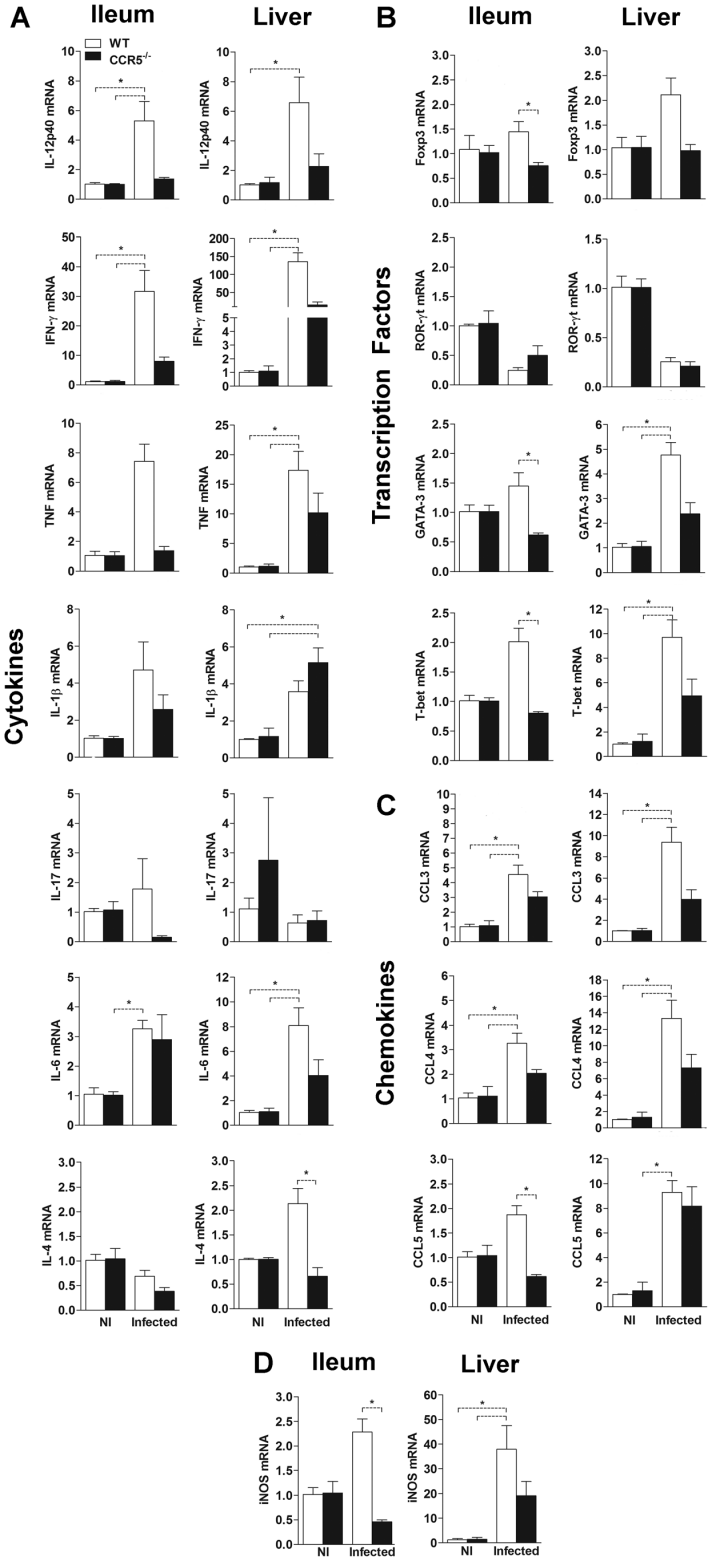
Imbalance in the immune response in CCR5^-/-^ mice infected with *T. gondii*. Expression of mRNA for the cytokines IL-12p40, IFN-γ, TNF, IL-1β, IL-17, IL-6 and IL-4 (A), transcription factors Foxp3, ROR-γt, GATA-3 and T-bet (B), chemokines CCL3, CCL4, CCL5 (C) and the enzyme iNOS (D) were determined by quantitative real time PCR (qPCR) of liver and ileum tissues collected on day 8 pi from WT and CCR5^-/-^ mice orally infected with 5 cysts of *T. gondii*. Data were normalized to GAPDH and Ct values were analyzed by 2^-ΔΔCt^ method, as shown in Materials and Methods. The data represent the mean ± SEM of results from three to five mice per group and are representative of two independent experiments. NI: non-infected. * p<0.05.

Because CCR5^-/-^ mice are more susceptible to *T. gondii* infection, we next assessed whether the absence of CCR5 affected the production of pro- and anti-inflammatory cytokines involved in the infection control. It was observed an increase in the transcripts of Th1 and inflammation-related cytokines IL-12p40, IFN-γ, TNF and IL-6 in the ileum and liver of infected mice, although in the absence of CCR5 the production of these cytokines seemed to be partially abolished (Figure 2A). Interestingly, the expression of IL-1β was increased in the ileum and liver of both infected groups but there was no difference between them. The mRNA of IL-17 was not differentially expressed among all groups, in spite of infection (Figure 2A). In addition, IL-4, a cytokine related to Th2 responses, was increased only in the liver of WT mice post infection (Figure 2A). These results suggested that in the absence of CCR5, the inflammatory response as well as the immune regulation induced by infection with *T. gondii* is compromised thus leading to a reduced parasite control. Then, to further investigate this hypothesis and to identify the putative predominant response in the ileum and liver of infected mice, we evaluated the mRNA expression of key transcription factors involved in the balance of the effector immune response. There was an augmented expression of the regulatory (Foxp3) and Th2 (GATA-3) transcription factors after *T. gondii* infection only in WT mice, while no difference was observed in the expression of the Th17-related nuclear receptor RORγt post infection, between WT and CCR5^-/-^ mice (Figure 2B). Most importantly, in the absence of CCR5, mice were not able to efficiently increase the expression of the Th1 transcription factor T-bet, essential to mounting an IFN-γ dependent response for parasite control, nor in the ileum or in the liver at 8 days post infection (Figure 2B). In agreement with these results, there was an apparent decreased expression of the CCR5 ligand chemokines CCL3 and CCL4 in the ileum and liver of CCR5^-/-^ infected animals and of CCL5 in the ileum, when compared to WT infected mice, indicating that the reduced levels of inflammatory cytokines and transcription factors could be accompanied by a compromised accumulation of leukocytes, in both sites of infection (Figure 2C). These statements were supported by the diminished expression of iNOS mRNA in the ileum and liver of infected mice, suggesting an impairment of the microbicidal activity in the parasite target tissues (Figure 2D).

### Infection with *T. gondii* induces liver damage, lipid change and hepatic steatosis in the absence of CCR5

**Figure 3:**
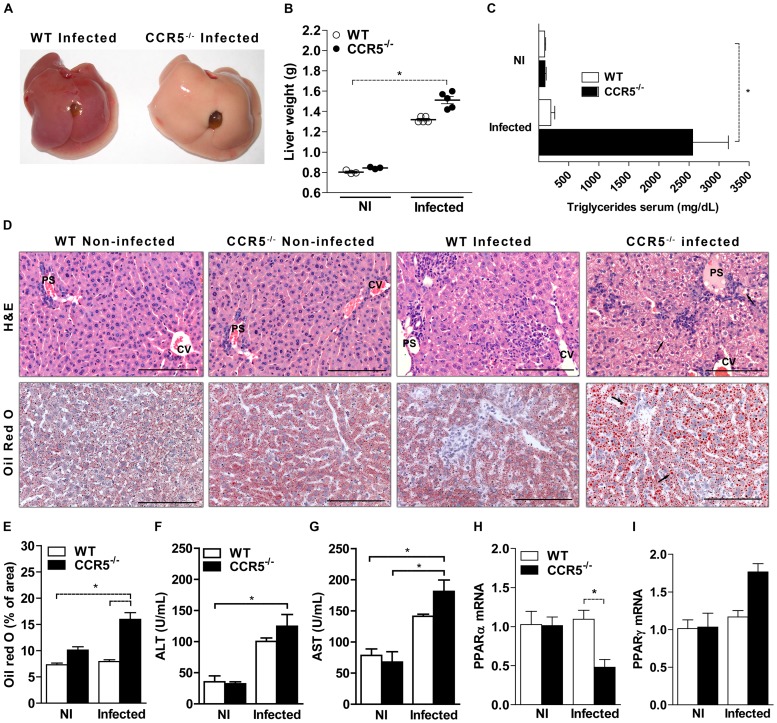
Lipid alterations and liver damage in CCR5^-/-^ infected mice. WT and CCR5^-/-^ mice were infected with 5 cysts of *T. gondii* and at day 8 pi the liver was harvested, photographed (A), weighed (B) and formalin-fixed for Hematoxylin and Eosin (H&E) staining (D, upper pannel) or frozen in OCT medium for Oil Red O staining (D lower pannel) and analysis by light microscopy. Lipid quantification was performed using specific software and expressed as percentage of area (E). Blood was collected and processed to serum triglycerides quantification (C), alanine aminotransferase-ALT (F) and aspartate aminotransferase-AST (G). The liver was also harvested in Trizol reagent, homogenized and total RNA extracted. Expression of PPARα (H) and PPARγ (I) was determined by quantitative real time PCR (qPCR). The data represent the mean ± SEM of results from three to five mice per group and are representative of two independent experiments. * p<0.05 compared to WT mice. NI: non-infected. PS: portal space, CV: centrolobular veins. Arrows: hepatocyte vacuolization. Scale bar: 100 µm.

Besides the differential immune response in both ileum and liver of CCR5^-/-^ mice, the necropsy appearance of CCR5^-/-^ infected liver directed our efforts to unraveling the role of this organ in the increased susceptibility to *T. gondii* infection. The liver of infected CCR5^-/-^ mice was macroscopically pale, with apparent larger size and increased weight, although no significant differences were observed when compared to WT infected mice (Figures 3A and B). The results of histopathological analysis showed that in both CCR5^-/-^ and WT non-infected controls this organ had normal appearance (Figure 3D, upper panel). However, the liver of WT and CCR5^-/-^ infected animals showed diffuse inflammatory foci both around centrolobular veins (CV) and portal spaces (PS), mainly composed by neutrophils associated with necrosis of hepatocytes (Figures 3D, upper panel). Most importantly, there was extensive hepatocyte vacuolization in CCR5^-/-^ mice that appeared as clean vacuoles within hepatocyte cytoplasm around the nuclei with clean borders that was suggestive of fatty change as a result of an important metabolic alteration (Figure 3D, arrows).

In fact, in the absence of CCR5, infected mice also showed elevated serum triglycerides compared to the other experimental groups (Figure 3C) and the mean area (in percentage) of stained lipid droplets was markedly increased in the liver of CCR5^-/-^ infected mice as compared to infected or non infected WT animals (Figures 3D lower panel and 3E). In addition, CCR5^-/-^ infected mice showed an increase in the concentration of ALT (alanine aminotransferase) and especially AST (aspartate aminotransferase) compared to uninfected groups (Figures 3F and G). In association with histological changes, these data indicated that the absence of CCR5 represented an extensive framework of liver damage, characteristic of steatosis, induced by the infection with *T. gondii*. Since infection with *T. gondii* in the absence of CCR5 led to an intense immunological and mainly metabolic injury of the liver, we next assessed the expression of transcription factors required for immune commitment or lipid metabolism in the liver. It was observed that at day 8 pi CCR5^-/-^ mice presented a lower expression of PPARα and no difference in PPARγ expression in the liver, when compared to WT infected mice (Figure 3H and 3I). Altogether, these results suggested that in the absence of CCR5 there is an important liver metabolic dysfunction and the liver failure could be a contributing factor to the death of the animals in the acute phase of infection with *T. gondii*.

### PPARα agonist does not restore the lipid metabolic alterations and does not rescue CCR5^-/-^ infected mice from death

**Figure 4:**
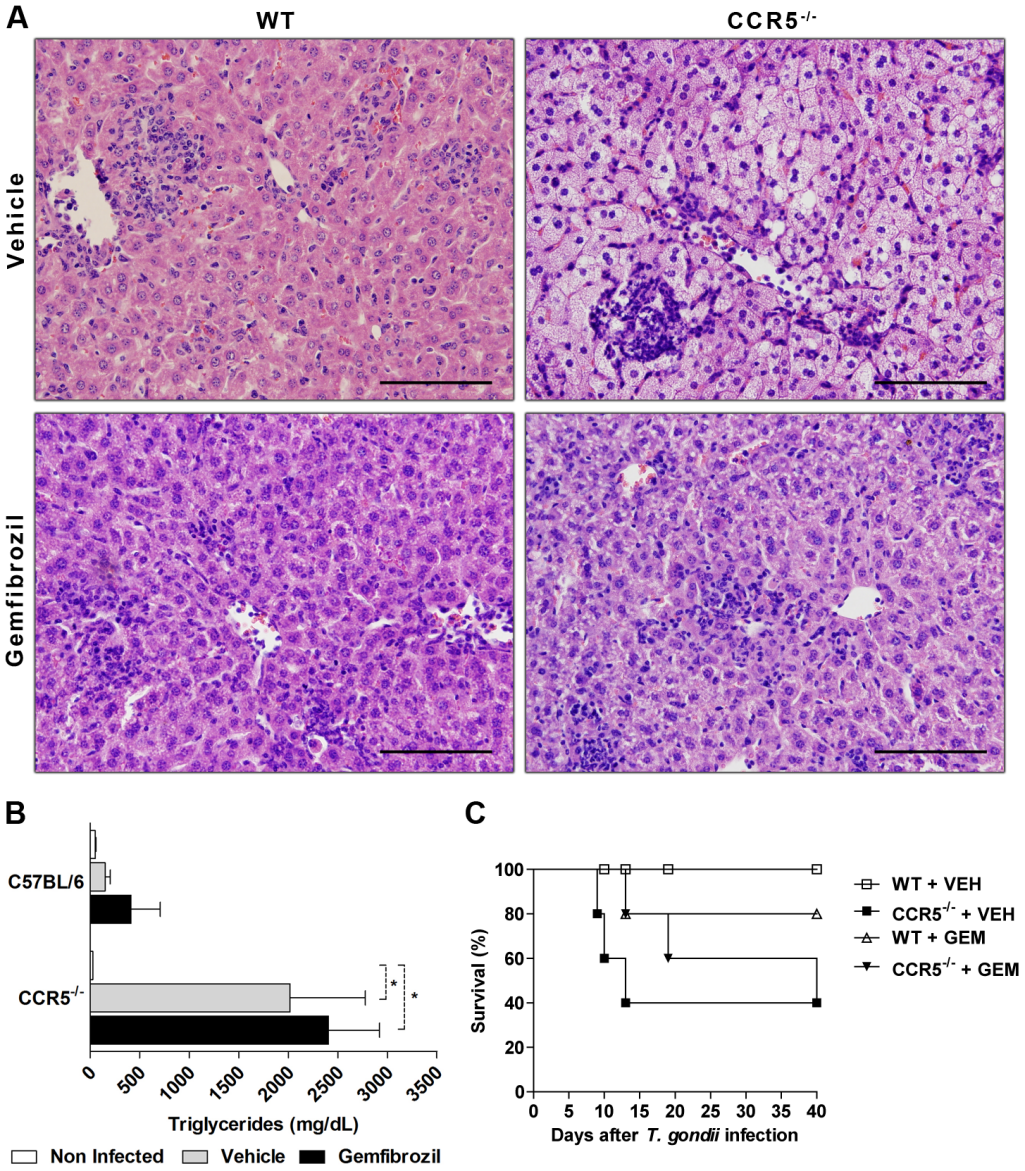
PPARα agonist partially restores liver damage but does not rescue CCR5^-/-^ infected mice from mortality. WT and CCR5^-/-^ mice were infected with 5 cysts of *T. gondii* and treated with Gemfibrozil, 100 mg/Kg/day, during 7 days. At day 8 pi, livers were harvested, formalin-fixed, paraffin-embedded, stained with Hematoxylin and Eosin (H&E) and analyzed by light microscopy (A). Blood was collected and processed to serum analysis of triglycerides (B). Survival was accompanied until day 40 pi and treatment. The results were obtained with three to five animals per group and are representative of two independent experiments. GEM: Gemfibrozil; VEH: vehicle. * p<0.05 compared to WT mice. Scale bar: 100 µm.

In an attempt to restore the metabolic alterations that could be leading to death in *T. gondii* infection in the absence of CCR5^-/-^, mice were treated with Gemfibrozil, a PPARα agonist used in the treatment of dyslipidemia. It was observed that, after 8 days of *T. gondii* inoculum and Gemfibrozil treatment, CCR5^-/-^ infected mice presented a reduction in liver vacuolization, suggestive of diminished lipid accumulation in the organ, thus resembling WT infected mice (Figure 4, lower panel). However, the PPARα agonist was not able to reduce serum triglycerides, liver inflammatory infiltrate or rescue CCR5^-/-^ infected mice from mortality (Figure 4A-C). Together, these results suggested that the activation of PPARα was only partially effective in the control of the hepatic damage, indicating that additional mechanisms could be involved in the increased susceptibility of CCR5^-/-^ mice to *T. gondii* infection.

### Increased susceptibility of CCR5^-/-^ mice to *T. gondii* is related to the intestinal injury

**Figure 5:**
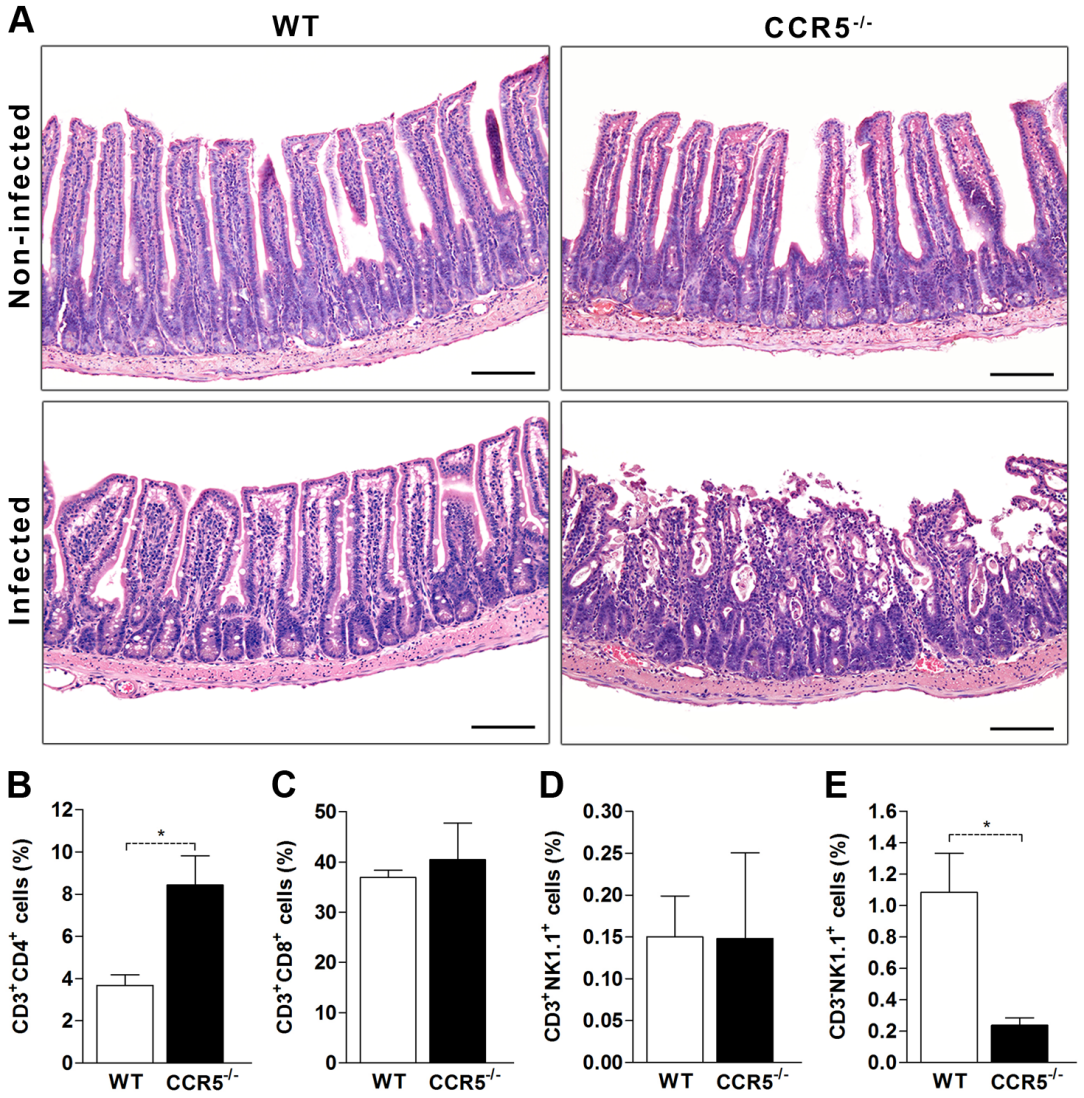
*Toxoplasma gondii* leads to inflammation, cellular infiltrate and ileum necrosis in the absence of CCR5. WT and CCR5^-/-^ mice were infected with 5 cysts of *T. gondii* and at day 8 pi, small intestine was harvested, formalin-fixed, paraffin-embedded, stained with Hematoxylin and Eosin (H&E) and analyzed by light microscopy (A). The leukocytes were isolated from lamina propria of small intestine of WT and CCR5^-/-^ mice at day 8 after *T. gondii* infection and characterized by flow cytometry. The percentage of CD3^+^CD4^+^ T (B), CD3^+^CD8^+^ T (C), CD3^+^NK1.1^+^ (D) and CD3^-^NK1.1^+^ (E) cells was evaluated by FlowJo software. Data represent the mean ± SEM of results from three to five mice per group and are representative of two independent experiments. NI: non-infected. * p<0.05 compared to WT mice.

Since liver damage was not directly associated to mice death, we sought to determine if an increased ileitis would be leading to the augmented susceptibility to infection in the absence of CCR5, once it is already known that WT C57BL/6 mice are highly susceptible to intestinal damage induced by *T. gondii* infection. The examination of the intestine of both non-infected control groups revealed the absence of inflammatory signs and intact integrity of the epithelial layer (Figure 5A, upper panel). As expected, WT infected mice presented a mild inflammatory change in the lamina propria (LP), epithelium and submucosa of the small intestine, composed by mononuclear cells with integrity of the epithelial barrier. In some areas there was a reduced length and increased thickness of the villi, accompanied by visible edema (Figure 5A, lower panel). Nevertheless, *T. gondii*-infected CCR5^-/-^ mice showed an intense inflammatory infiltrate in the small intestine, predominantly in the jejunum (data not shown) and ileum, which was visualized as a complete disorganization of the villi with necrotic foci (Figure 5A, lower panel). This intense ileitis was associated with focal and diffuse destruction of the epithelial barriers. In some areas there were bounded regions of epithelial ulceration. The inflammatory response appeared to be associated with hemorrhage in the small intestinal wall as well as a few mononuclear cells. These leukocytes seemed to be composed by an increased accumulation of CD3^+^CD4^+^ cells in the absence of CCR5 when compared to WT (Figure 5B), while no difference was observed in the frequency of CD3^+^CD8^+^ or CD3^+^NK1.1^+^ cells (supposedly NKT cells, Figures 5C and D). However, in WT infected mice there was an augmented frequency of NK cells in the ileum lamina propria, which may have accounted for helping in parasite elimination, a fact that did not occur in CCR5^-/-^ infected mice, which behave similarly to non-infected animals regarding the accumulation of this cell population in the small intestine (Figure 5E and data not shown). Most interestingly, we found an apparent intense inflammatory infiltration of neutrophils in the small intestine of CCR5^-/-^ mice after *T. gondii* infection.

### The absence of CCR5 leads to neutrophils accumulation after *T. gondii* infection

**Figure 6:**
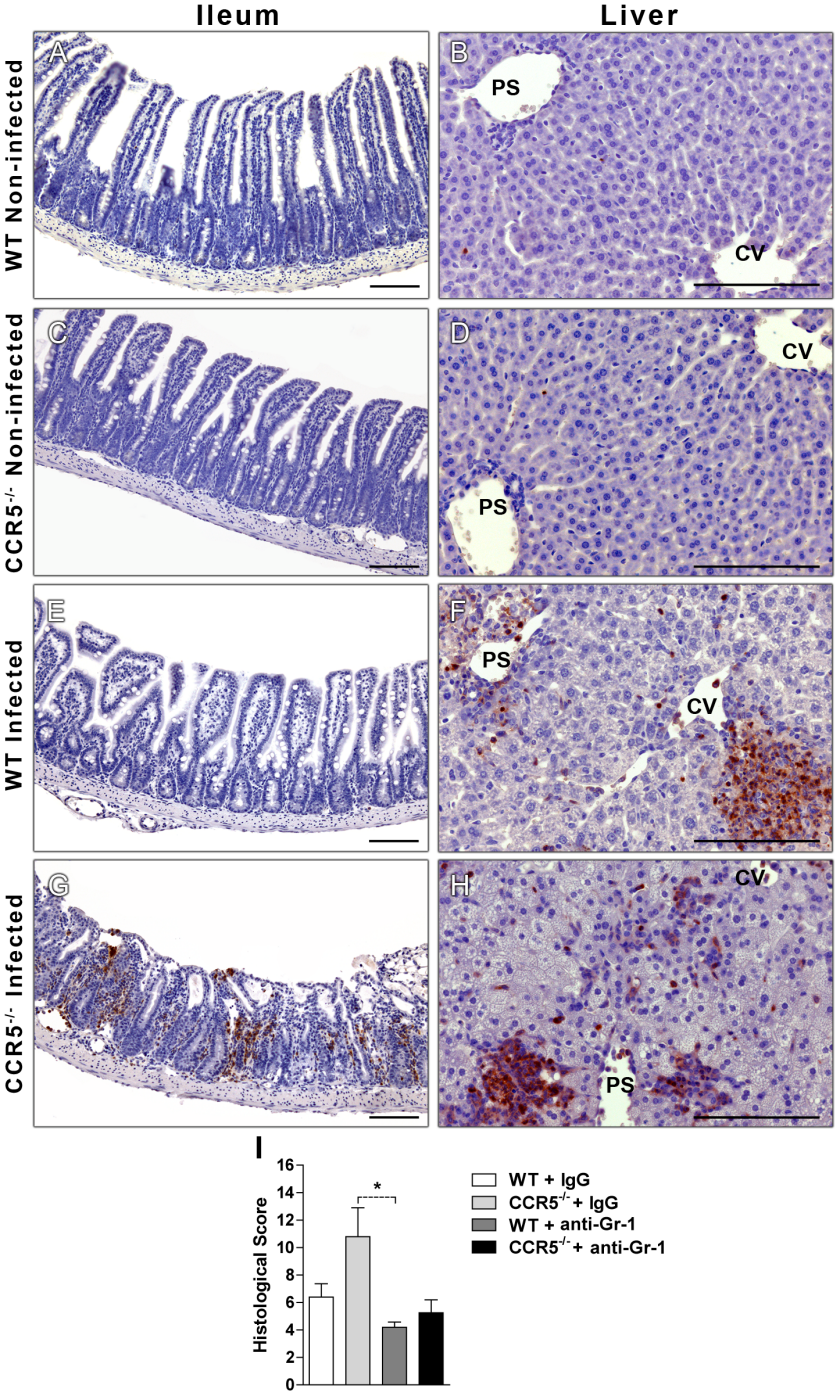
Increased accumulation of neutrophils and tissue damage in CCR5^-/-^ infected mice. WT and CCR5^-/-^ mice were infected with 5 cysts of *T. gondii* and at day 8 pi the ileum and liver were harvested, formalin-fixed and paraffin-embedded. Sections were stained with a specific anti-neutrophil antibody (α-Ly6B) and analysis was made by light microscopy (A-H). WT and CCR5^-/-^ mice were infected, depleted with anti-Gr-1 antibody (RB6-8C5) and at day 8 pi the small intestine was harvested, formalin-fixed, paraffin-embedded, stained with Hematoxylin and Eosin (H&E) and analyzed by light microscopy (I). The histological score comprised the sum of the following parameters, for each mouse: intensity of lamina propria (LP) inflammatory infiltration; thickening of LP; destruction of the villi and necrosis, in a scale that ranged from 0 to 5 of intensity. Data were obtained with three to five animals per group and experiments were performed twice with similar results. PS: portal space, CV: centrolobular veins. Scale bar: 100 µm.

Once histological analysis suggested an increased presence of neutrophils in CCR5^-/-^ infected mice, not only ileum but also liver sections were stained with an anti-neutrophil antibody (anti-Ly6B) for immunohistochemistry evaluation of the presence of these granulocytes and their association to *T. gondii* related tissue injury. We observed that CCR5^-/-^ infected mice had an increased number of neutrophils in the ileum, when compared to WT infected animals, which showed a complete clearance of these cells at day 8 post-infection (Figures 6E and 6G). In accordance to the histological analysis, there was increased number of neutrophils in the liver of both infected groups in comparison to non-infected mice (Figures 6B, D, F and H) and no specific difference was observed in the accumulation of these cells between the liver of WT and CCR5^-/-^ infected mice. Most interestingly, the depletion of neutrophils with anti-Gr-1 antibody seemed to reduce inflammation and tissue damage in the CCR5^-/-^ infected mice as compared to those that were treated with rat IgG, in the absence of this chemokine receptor (Figure 6I). Therefore, these results indicated that the absence of CCR5 is related to neutrophil accumulation especially in the small intestine and this cell type may be related to the intense inflammatory lesions and ulcerations in the ileum, along with increased susceptibility to *T. gondii* infection in CCR5 knockout mice.

## Discussion

The immune response against *T. gondii* infection involves activation of mechanisms of innate and adaptive immunity after parasite invasion of enterocytes in the small intestine, followed by the production of soluble factors, like chemokines, that recruit cells able to kill the parasite. In this study we found that in the absence of CCR5 mice succumb to infection with uncontrolled parasite growth, altered lipid metabolism, hepatic steatosis and extensive intestinal damage characterized by ileum necrosis with a prominent neutrophils infiltrate.

The early immune response against the parasite involves the recruitment of various cell types such as T cells, neutrophils, inflammatory monocytes, macrophages and dendritic cells [22], [34] mediated by some chemokines produced by infected intestinal epithelial cells. Considering our results of augmented CCR5 expression and its ligands CCL3, CCL4 and CCL5 post infection, it is possible to suggest that there was an increased recruitment of CCR5^+^ cells to the parasite target organs or resident cells were able to up regulate the expression of these molecules during the infection with *T. gondii* in WT mice. Indeed, CCR5 is expressed by resident cells or by those that migrate to the tissue after stimulation with pro-inflammatory cytokines (IL-12, IFN-γ and TNF) upon contact with pathogens [35]. Moreover, CCL3 is expressed by NKp46+ innate lymphoid cells in response to inflammatory cytokines (like IL-18) and recruit CCR1+ inflammatory monocytes to the site of *T. gondii* infection [36]. Along with increased expression of chemokine receptors in WT infected animals, we found that CCR5^-/-^ mice had a higher parasite load associated with elevated mortality compared to WT infected mice. In contrast to our data, other studies showed that CCR5^-/-^ mice are able to control infection by *Mycobacterium tuberculosis* [37], *Listeria monocytogenes* [38] and also decrease the development of cerebral malaria, when animals were infected with *Plasmodium berghei* ANKA [39]. On the other hand, previous studies also showed that CCR5^-/-^ mice are more susceptible to infection with *T. gondii*, with a higher parasite load and decreased production of IL-12 during acute infection by this parasite [4], [13]. Thus, the uncontrolled parasite growth and infection susceptibility could be partially associated to altered cell function due to CCR5 absence, once we found increased parasitism and cellular infiltrate, together with an overall reduced expression of cytokines and microbicidal molecules in CCR5^-/-^ infected mice.

Certainly, cells that can be recruited via CCR5 are partly responsible for the mechanisms that control the parasite and the subsequent inflammation. During the infection, DC are recruited to the site of inflammation in response to CCR5 ligands produced by neutrophils and enterocytes, while the CCR5 antagonist Met-RANTES partially impairs STAg-induced DC migration, as a result of impediment of cells to be exposed to autocrine CCR5 ligands [4]. These DC are so important in the production of IL-12 that are essential to resistance against infection [40]. Similarly, in agreement with our results, NK cells with increased activity [41] are the major source of IFN-γ early in infection [42] and their recruitment to the intestine is related to CCR5 [13]. Here, we observed a partial reduced expression of pro-inflammatory cytokines IL-12p40, IFN-γ and TNF in the ileum and liver of CCR5^-/-^ mice compared to WT infected ones. This reduction in cytokines that induce microbicidal effect, may be related to the important role that CCR5 plays in inducing the synthesis of IL-12 after stimulation with parasite secreted protein cyclophilin 18 [4], [25], thus accounting to the uncontrolled immune response and high parasitism in the ileum and liver, as observed in the present work. Notably, the expression of iNOS, one of the enzymes responsible for NO production, was also diminished in the absence of CCR5, indicating that this chemokine receptor also interferes in one of the main parasite microbicidal mechanisms [43]. Furthermore, our study also showed decreased expression of the chemokines CCL3, CCL4 and CCL5 in the liver and ileum of CCR5^-/-^ mice along with reduced induction of the transcription factors Foxp3, T-bet and GATA-3 after infection. These data demonstrated that most immune response patterns, in special the Th1 necessary for the parasite control, are impaired in the absence of CCR5.

After infecting the intestinal cells, the parasite spreads rapidly through the lymphatic vessels to lymph nodes, liver and blood or from the liver to the lungs and other organs. The induction of the common CCR5 ligands was more evident in the liver compared to the ileum and this may indicate the marked importance of this chemokine-receptor interaction in liver tissue. Interestingly, the observation of white aspect of the liver and lipidemic serum in CCR5^-/-^ infected mice led us to associate mortality with liver injury and look for these putative hepatic alterations. In fact, along with high parasitism, CCR5^-/-^ mice also presented serious tissue damage in combination with intense inflammatory infiltrate and high accumulation of fatty acids in the liver, possibly related to the high concentration of triglycerides and transaminases in sera. These data suggested a putative metabolic disorder resulting from the absence of CCR5 in combination with the inflammatory response induced by infection. Some studies have suggested a possible relationship between CCR5 absence and the progression of liver disease [44], [45]. Regarding hepatitis C, the homozygous 32-base pair deletion in the CCR5 gene (CCR5-Δ32), associated to protection against HIV, is more predominant in hepatitis C virus (HCV) patients than in healthy controls, suggesting that CCR5 mutation can be a factor that predisposes to hepatic infection [44]. Moreover, the absence of CCR5 may also lead to exacerbation of T cell-mediated hepatitis in mice, dependent on the production of TNF [45]. Regardless cellular recruitment, the absence of the chemokine receptor CCR5 leads to a condition known as fulminant liver failure (FLF) when concanavalin A (Con A) is administered to experimental animals. This condition occurs by activation of CD1d restricted NKT cells with increased production of IL-4 and IFN-γ [46], in contrast to our results that showed a decreased expression of IFN-γ and IL-4 in the liver of CCR5^-/-^ *T. gondii-*infected mice. Moreover, the liver is an important source of TGF-β and IL-6, that are, together with IL-1β, responsible to induce differentiation of the Th17 phenotype [47], [48]. In our data, the increased IL-1β in the liver of CCR5^-/-^ infected mice did not apparently converge to Th17 differentiation, as we observed decreased IL-6 and ROR-γt transcripts together with no difference in the IL-17 expression. Therefore, in view of the literature and the results described herein, we could state that the absence of CCR5 together with *T. gondii* infection led to an impairment of the liver function that could comprise host systemic metabolism and inflammation. Indeed, one of known pathways involved in lipid metabolism are related to PPARα and γ receptors.

Peroxisome proliferator-activated receptors (PPAR) are transcription factors activated by ligand, belonging to the superfamily of nuclear receptors that includes steroid hormone, vitamin D receptor, retinoic acid (RA) and thyroid hormone receptors [49], [50]. Our gene expression data pointed to decreased expression of PPARα in the liver of CCR5^-/-^ infected mice at the 8th day after infection. PPARα is known to regulate fatty acid catabolism and to increase the transcription of genes involved in intracellular transport of fatty acids to peroxisomes and mitochondria for β-oxidation [50]. These data, together with the extensive framework of hepatic vacuolization and accumulation of lipids in the liver, suggested that the infection with *T. gondii* in the absence of CCR5 may have led to the block or a to lack of activation of some metabolic pathways involving peroxisome or mitochondria, responsible for β-oxidation or lipid neogenesis, induced by PPARα.

PPARα is the target of an agonist class of drugs known as fibrates such as Gemfibrozil and Fenofibrato. These drugs are commonly used clinically to treat hypertriglyceridemia, besides being safe and well tolerated by patients [51]-[54]. By treating animals infected with *T. gondii* with PPARα agonist, Gemfibrozil, CCR5^-/-^ mice showed improvement in the vacuolization of liver, but no change in serum triglycerides levels or mortality. These results suggested that PPARα could be related to the mechanism of steatosis and liver damage in CCR5-deficient animals but was not the main cause of mice mortality after *T. gondii* infection. Thus, the primary effect of the absence of CCR5 was probably the inhibition of mitochondrial β-oxidation and in the course of infection by *T. gondii* it led to liver damage as evidenced by steatosis.

Since mice did not seem to succumb to infection essentially due to liver damage, we evaluated the ileum as the main target of *T. gondii* induced pathology. In fact, the gut of CCR5^-/-^ infected mice presented extensive lesions with elevated frequency of CD4 T lymphocytes and reduced NK cells. It is known that CD4^+^ T cells producing IFN-γ cells are the major mediators of inflammatory response to infection by *T. gondii* [20], although in the absence of CCR5, the accumulation of CD3^+^CD4^+^ T cells did not seem to be enough to constrain parasite replication in the gut. It is possible that these CD4 T lymphocytes were not IFN-γ producers or even the low frequency of NK cells might have corroborated to the low levels of this phagocyte activation cytokine. In fact, NK cells present a role in the early resistance to *T. gondii* as a source of IFN-γ and are supposed to recognize and kill infected cells [42]. Moreover, NK cell activity is increased after *T. gondii* infection, showing their important role in parasite control [41]. Although the adaptive immune response was affected in CCR5^-/-^ infected mice, an increased compensatory accumulation of neutrophils was observed in the ileum and liver of these animals. Thus, the lack of clearance of these cells together with a putative augmented activity of neutrophils *in situ*, in an attempt to combat the parasite, may have resulted in the extensive tissue damage and even mice death. Nevertheless, although neutrophils may be partially responsible for IFN-γ production and parasite control during *T. gondii* infection [55], in our experiments we observed opposite results, indicating that the accumulation of these cells in the absence of CCR5 was not linked to augmented cytokine production or parasite elimination. In addition, other neutrophil-derived molecules may be responsible for intestinal damage and this cell may play different roles during infection. Accordingly, CCR2^-/-^ mice orally infected with *T. gondii* presented a deficient recruitment of Gr-1^+^ inflammatory monocytes, uncontrolled parasite replication, intestinal necrosis, rapid death and it was also associated to an extensive infiltration of neutrophils [34]. In other model, neutrophils are associated to acute intestinal inflammation when recruited after TLR2 and TLR4 activation [56]. Indeed, neutrophils expressing elastase, myeloperoxidase and proteinases MMP-9 were found in the tissue-damaged areas of mucosal leishmaniasis patients [57], suggesting that accumulation of these cells could be associated to tissue lesions. On the other hand, ablation of the recruitment of neutrophils in CXCR2^-/-^ mice induced production of IFN-γ and TNF but only mild susceptibility to *T. gondii* acute infection in mice [58], despite the evidence that neutrophils produce IL-12 in response to this parasite [59]. In fact, the ileum damage in our model of *T. gondii* infection seemed to be related to neutrophils accumulation since depletion of these cells with anti-Gr-1 antibody resulted in less evident lesions and decreased inflammation in CCR5^-/-^ infected and treated mice. However, it should be noted that other cell populations could also play a minor role in the increased susceptibility to gut damage in the absence of CCR5, once the RB6-8C5 antibody used here depletes cells expressing Gr-1 molecule (Ly6C^+^ and Ly6G^+^) which may be expressed by a subset of monocytes too [60].

Finally, our study demonstrated that CCR5 is essential to the control of *T. gondii* infection. The absence of this receptor leads to a high susceptibility to the parasite once a metabolic and immune dysfunction occur simultaneously and impair the infection clearance that would be expected from an immune competent host. These findings add novel information on the disease pathogenesis and may be relevant for directing future approaches to the treatment of multi-deregulated inflammatory or infectious diseases.
